# Opioid prescribing practices at hospital discharge for surgical patients before and after the Centers for Disease Control and Prevention’s 2016 opioid prescribing guideline

**DOI:** 10.1186/s12871-022-01678-6

**Published:** 2022-05-11

**Authors:** Erica Langnas, Andrew Bishara, Rhiannon Croci, Rosa Rodriguez-Monguio, Elizabeth C. Wick, Catherine L. Chen, Zhonghui Guan

**Affiliations:** 1 Department of Anesthesia and Perioperative Care, University of California San Francisco, 513 Parnassus Ave, S455, San Francisco, CA 94143 USA; 2 UCSF Health Informatics, University of California San Francisco, San Francisco, CA USA; 3 Philip R. Lee Institute for Health Policy Studies at UCSF, San Francisco, CA USA; 4 Department of Clinical Pharmacy, University of California San Francisco, San Francisco, CA USA; 5 Medication Outcomes Center, University of California San Francisco, San Francisco, CA USA; 6 Department of Surgery, University of California San Francisco, San Francisco, CA USA

**Keywords:** Oral morphine equivalents, Opioids, Post-operative pain, Health policy

## Abstract

**Background:**

The Centers for Disease Control and Prevention’s (CDC) March 2016 opioid prescribing guideline did not include prescribing recommendations for surgical pain. Although opioid over-prescription for surgical patients has been well-documented, the potential effects of the CDC guideline on providers’ opioid prescribing practices for surgical patients in the United States remains unclear.

**Methods:**

We conducted an interrupted time series analysis (ITSA) of 37,009 opioid-naïve adult patients undergoing inpatient surgery from 2013–2019 at an academic medical center. We assessed quarterly changes in the discharge opioid prescription days’ supply, daily and total doses in oral morphine milligram equivalents (OME), and the proportion of patients requiring opioid refills within 30 days of discharge.

**Results:**

The discharge opioid prescription declined by -0.021 (95% CI, -0.045 to 0.003) days per quarter pre-guideline versus -0.201 (95% CI, -0.223 to -0.179) days per quarter post-guideline (*p* < 0.0001). Likewise, the mean daily and total doses of the discharge opioid prescription declined by -0.387 (95% CI, -0.661 to -0.112) and -7.124 (95% CI, -9.287 to -4.962) OME per quarter pre-guideline versus -2.307 (95% CI, -2.560 to -2.055) and -20.68 (95% CI, -22.66 to -18.69) OME per quarter post-guideline, respectively (*p* < 0.0001). Opioid refill prescription rates remained unchanged from baseline.

**Conclusions:**

The release of the CDC opioid guideline was associated with a significant reduction in discharge opioid prescriptions without a concomitant increase in the proportion of surgical patients requiring refills within 30 days. The mean prescription for opioid-naïve surgical patients decreased to less than 3 days’ supply and less than 50 OME per day by 2019.

**Supplementary Information:**

The online version contains supplementary material available at 10.1186/s12871-022-01678-6.

## Introduction

Post-surgical prescriptions are a major source of hospital opioid prescriptions at discharge [1]. In the United States, opioids are often over-prescribed to patients after surgical procedures [2–8]. As a result, excess opioids can be a potential source for overdose, misuse, diversion, and new persistent opioid use among surgical patients [8–14]. There is a general consensus among medical providers that postoperative opioid prescriptions should be optimized to balance the provision of effective postoperative pain control and the risk of opioid-related adverse effects [9–11].

In response to the opioid prescription epidemic, the Centers for Disease Control and Prevention (CDC) of the United States published the *Guideline for Prescribing Opioids for Chronic Pain*in March 2016 to improve the safety and effectiveness of pain treatment and to reduce the risks of opioid use disorder, overdose, and death [12]. The guideline recommended prescribing up to three days’ supply for patients with non-surgical acute pain, and suggested a careful assessment of risks and benefits when the prescribed dose exceeds 50 oral morphine milligram equivalents (OME) per day [12]. Significant improvements in opioid prescribing practices in non-surgical patients have been observed after the release of 2016 CDC guideline [13].

It is important to note that the CDC guideline was not intended to address postoperative opioid prescribing [12], and the CDC has not yet developed a formal guideline to address opioid prescribing in postoperative patients. Sutherland et al. recently analyzed a private insurance database for patients undergoing 8 common surgical procedures and found an association of decreased opioid dispensing after the CDC guideline in opioid naïve surgical patients, which highlights the potential influence of these guidelines in the surgical setting [14]. However, this study focused on opioids that were filled by patients but did not include data on the opioid prescriptions that were written by providers [14]. Prior studies have found that up to 21% of opioids remain unfilled after surgery [4]. Therefore, it remains unclear if the trends they observed were the result of patients’ decision to fill less postsurgical opioid prescriptions, or the result of providers changing their opioid prescribing practices change after the CDC guideline.

To investigate the potential effect of 2016 CDC guideline on provider level opioid prescribing practice, we assessed the opioid prescribing practices for surgical patients upon discharge at a large academic medical center in California between 2013 and 2019 with interrupted time series analysis. We examined trends in the days’ supply, total and daily dose of the discharge opioid prescription, and 30-day opioid refill prescriptions written for opioid-naïve patients undergoing all inpatient surgical procedures.

## Materials and methods

### Study design and data source

We conducted an interrupted time series analysis (ITSA) of adult opioid-naïve patients undergoing inpatient surgery from January 2013 through December 2019 at University of California San Francisco Medical Center. Study data were derived from the electronic medical record (EMR) system (Epic Systems, Verona, WI). This study was approved by the IRB at our institution, which waived patient consent for acquisition of data (IRB# 18–26,728).

After extraction from an electronic data warehouse, the data were validated for accuracy with iterative chart auditing. To ensure accurate and complete data extraction, data reports were evaluated to identify inconsistencies, missingness, extreme values, and invalid codes. Discrepancy management included reviewing discrepancies, investigating the reason, and resolving them. The data extracted had no missingness. After a proper quality check and assurance, the final dataset was locked so that the dataset could not be modified and only the final clean dataset was used for analysis.

### Study cohort

Our study included all opioid-naïve patients aged 18 years and older who underwent any surgery requiring a post-operative inpatient stay of at least 24 h after leaving the post-anesthesia care unit (PACU), and who were discharged to either home, a skilled nursing facility or rehabilitation facility. If a patient had multiple surgical procedures during the same surgical admission, we used the first surgical procedure as the index procedure for that admission. If a patient had additional procedures during the study period during subsequent hospitalizations, we categorized those subsequent hospitalizations as new patient encounters. We excluded patients discharged to other acute level care sites and hospice. We defined opioid-naïve as any patients without opioids listed on their medication list at the time of admission and without an active opioid prescription documented in their EMR starting six months prior to the date of surgery. Patients were stratified based on whether they underwent surgery before or after the release of the March 2016 CDC opioid guideline. Hence, patients who underwent surgery between the 1^st^ quarter of 2013 to the 1^st^ quarter of 2016 were included in the pre-guideline group and patients who underwent surgery between the 2^nd^ quarter of 2016 to the 4^th^ quarter of 2019 were included in the post-guideline group.

### Opioid dose calculation

The opioid dosage on the discharge opioid prescription was converted into OMEs using standard opioid conversion ratios [15]. The daily dose on the discharge opioid prescription was defined as the maximum allowable dose in a 24-h period according to the written prescription. Whenever more than one prescription was prescribed to the patient at hospital discharge, the daily OME and total OME on the opioid discharge prescription were calculated as the sum of all written opioid prescriptions. Opioid dose reflects the dose that was prescribed by providers at discharge and not what was filled by patients. Opioid refill prescriptions were defined as opioid prescriptions written after the discharge opioid prescription between 1 and 30 days after hospital discharge.

### Statistical analysis

We used descriptive statistics to compare baseline differences in patient characteristics in the.

pre- and post- guideline groups. The mean days’ supply, mean daily OME, and mean total OME at discharge, and the percentage of patients who required opioid refill prescriptions within 30 days of hospital discharge were assessed quarterly. Data were analyzed by segmental linear regression with least squares regression (Prism 9.0) as an interrupted time series analysis (ITSA). We reported the slopes and their 95% confident intervals (CI) before and after the CDC opioid guideline was released, as well as the *p*-values to determine the model as segmental linear regression or as simple linear regression of a straight line. Additional detail about the ITSA can be found in the Supplemental Material 1.

The differences between the 1^st^ quarter of 2016 and the last quarter of 2019 in day’s supply, daily and total doses were analyzed with unpaired two-tailed t test. The differences between the 1^st^ quarter of 2016 and the last quarter of 2019 in percentage of patients receiving more than 3 days’ supply or more than 50 OME per day, and the percentage of patients requiring opioid refill prescriptions within 30 days of discharge were analyzed with two-tailed Chi-square test. Both analyses were conducted with Prism 9.0.

## Results

A total of 37,009 opioid naïve patients undergoing any inpatient surgical procedures met inclusion criteria. There were 15,288 patients in the pre-guideline group, and 21,721 patients in the post-guideline group. Patient characteristics were similar between two groups on age, gender, race, length of stay, and mental health disorders (Table 1). There was no significant change in postsurgical hospital stay before or after the guideline. The proportion of surgical volume by service was similar between pre- and post-guideline, with the exception of obstetrics and gynecology due to the expansion of our women and children’s hospital in 2015 (Table 1).

**Table 1:** Patient demographic and clinical attributes pre- and post- 2016 CDC guideline

	Pre-guideline (*n* = 15,288)	Post-guidelines (*n* = 21,721)
Age, mean (SD)	55.58 (16.86)	54.967 (17.52)
Race, No. (%)		
White or Caucasian	9264 (60.60)	12,907 (59.42)
Asian	1956 (12.79)	3151 (14.51)
Black or African American	801 (5.24)	1202 (5.53)
Native Hawaiian or Other Pacific Islander	197 (1.29)	259 (1.19)
American Indian or Alaska Native	71 (0.46)	147 (0.68)
Unknown	2999 (19.62)	4055 (18.67)
Sex, No. (%)		
Male	7604 (49.738)	9891 (45.537)
Female	7683 (50.265)	11,827 (54.450)
Non-binary	1 (0.007)	2 (0.009)
Mental Health Disorders, No. (%)		
Anxiety	1496 (9.79)	1904 (8.77)
Depression	373 (2.44)	801 (3.69)
Substance Use Disorder	115 (0.75)	217 (1.00)
Primary Service, No. (%)		
Cardiac Surgery	459 (3.00)	613 (2.82)
Colorectal Surgery	362 (2.37)	510 (2.35)
General Surgery	1897 (12.41)	2865 (13.19)
Gynecologic Oncology	427 (2.79)	475 (2.19)
Kidney Transplant	1114 (7.29)	1022 (4.71)
Liver Transplant	406 (2.66)	791 (3.64)
Neurosurgery	3268 (21.38)	4033 (18.57)
Obstetrics and Gynecology	772 (5.05)	2422 (11.15)
Oral & Maxillofacial Surgery	112 (0.73)	211 (0.97)
Orthopedics	2137 (13.98)	3196 (14.71)
Otolaryngology, Head & Neck Surgery	571 (3.73)	909 (4.18)
Plastic Surgery	238 (1.56)	249 (1.15)
Thoracic Surgery	261 (1.71)	209 (0.96)
Urology	1710 (11.19)	1938 (8.92)
Vascular Surgery	572 (3.74)	637 (2.93)
Other	982 (6.42)	1641 (7.55)

### Trend change in days’ supply of postoperative discharge opioid prescriptions after CDC guideline

**Fig. 1:**
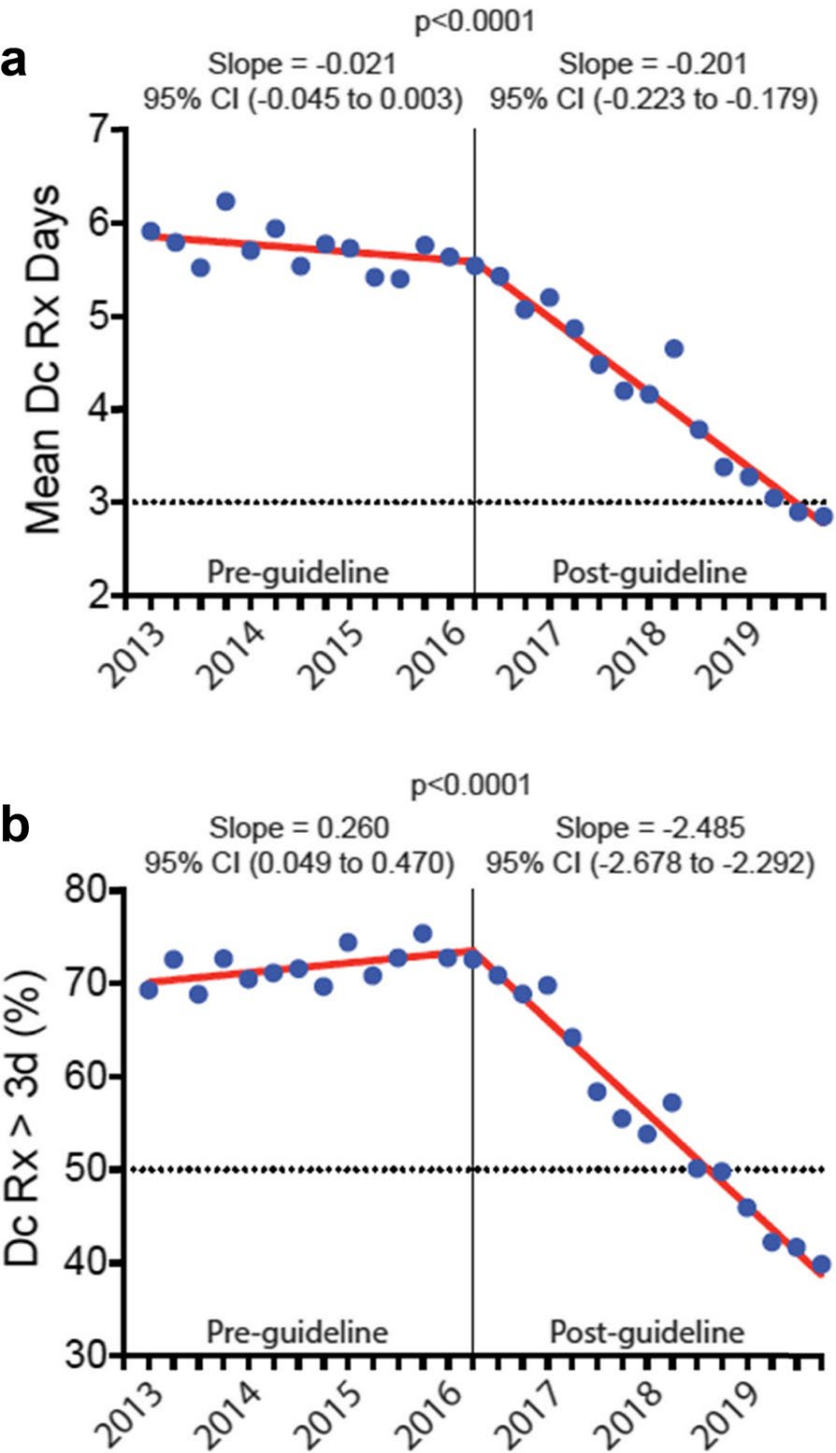
Duration of postoperative discharge opioid prescription, 2013–2019. **a** Mean duration of the discharge opioid prescription for postoperative patients before and after the release of the CDC opioid guideline. Data are presented as the mean days’ supply on the discharge opioid prescription in quarterly intervals from the 1^st^ quarter of 2013 through the 4^th^ quarter of 2019. The vertical line represents the first quarter after the release of the CDC opioid guideline in March 2016. By the 3^rd^ quarter of 2019, the mean duration of the postoperative discharge opioid prescription had decreased to less than 3 days. **b** Proportion of postoperative patients receiving a discharge opioid prescription with greater than 3 days’ supply before and after the release of the CDC opioid guideline. Data are presented as the percentage of postoperative patients receiving a discharge opioid prescription with greater than 3 days’ supply in quarterly intervals from the 1^st^ quarter of 2013 through the 4^th^ quarter of 2019. The vertical line represents the first quarter after the release of the CDC guideline in March 2016. By the 4^th^ quarter of 2018, the proportion of postoperative patients receiving a discharge opioid prescription with greater than 3 days’ supply had decreased to less than 50%. Dc = discharge; Rx = prescription, 3d = 3 days, and CI = confidence interval

The quarterly change in the mean days’ supply on the discharge opioid prescription was -0.021 days per quarter in the pre-guideline period (95% CI, -0.045 to 0.003) (Fig. 1a). In the post-guideline period, the trend changed significantly, with a quarterly change in mean days’ supply of -0.201 (95% CI, -0.223 to -0.179, *p* < 0.0001). Overall, the mean days’ supply decreased by 49.5%, from 5.64 ± 0.14 days in the 1^st^ quarter of 2016 to 2.85 ± 0.09 days in the last quarter of 2019 (*p* < 0.0001).

In the pre-guideline period, the proportion of patients who received a discharge opioid prescription of greater than 3 days’ supply increased by 0.260 percentage points per quarter (95% CI, 0.049% to 0.470%) (Fig. 1b). In the post-guideline period, the trend changed significantly, decreasing by 2.485 percentage points per quarter (95% CI, -2.678% to -2.292%, *p* < 0.0001). Overall, the proportion of patients who received a discharge opioid prescription of greater than 3 days’ supply decreased by 45.3%, from 72.8% in the 1^st^ quarter of 2016 to less than 39.8% in the last quarter of 2019 (*p* < 0.0001).

### Trend change in daily dose of postoperative discharge opioid prescriptions after CDC guideline

**Fig. 2:**
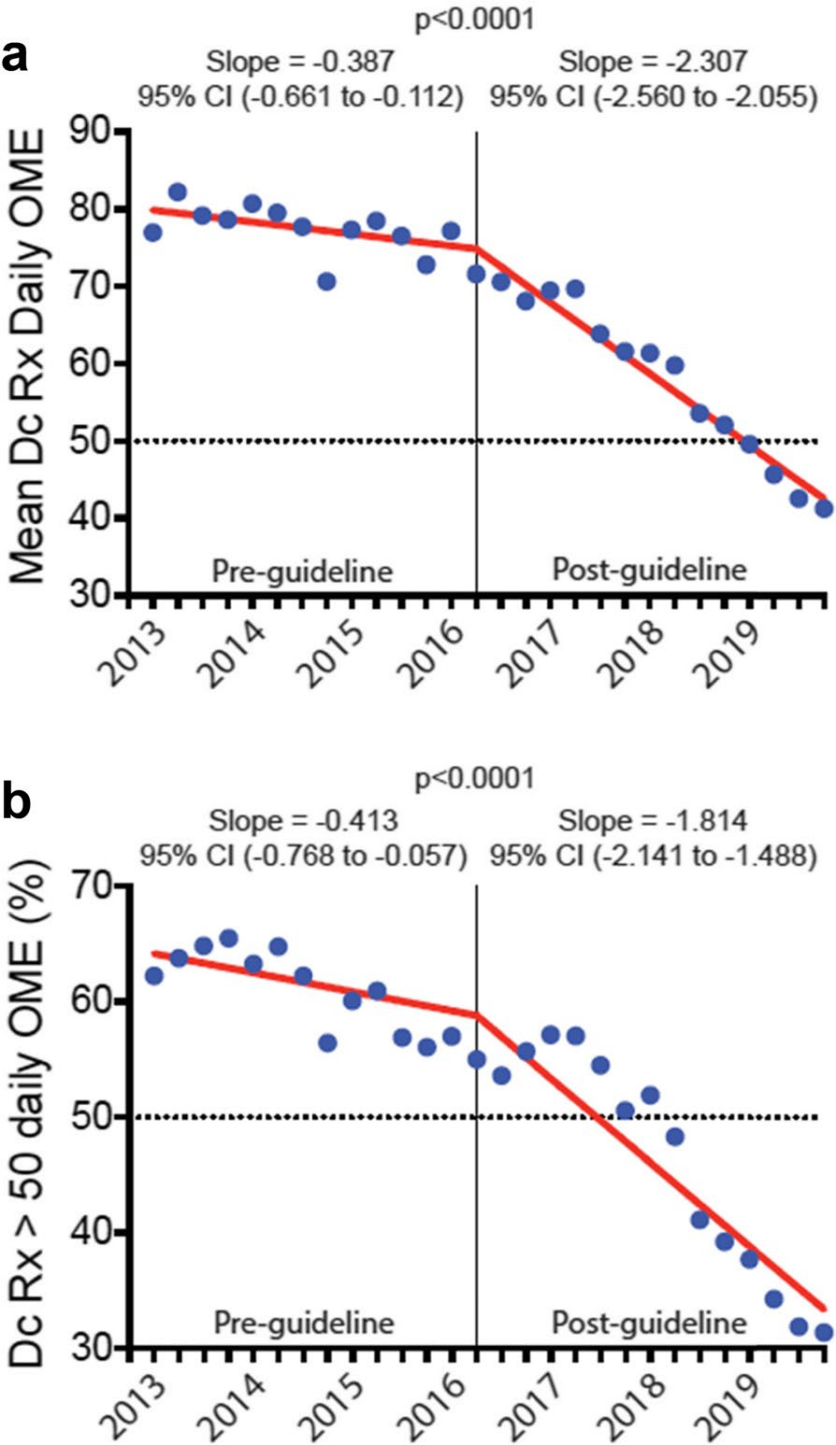
Daily dose on the postoperative discharge opioid prescription, 2013–2019. **a** Mean daily dose prescribed on the postoperative discharge opioid prescription before and after the release of the CDC opioid guideline. Data are presented as the mean daily dose prescribed on the postoperative discharge opioid prescription in quarterly intervals from the 1^st^ quarter of 2013 through the 4^th^ quarter of 2019. The vertical line represents the first quarter after the release of the CDC guideline in March 2016. By the 1^st^ quarter of 2019, the mean daily dose on the postoperative discharge prescription had decreased to less than 50 OME. **b** Proportion of postoperative patients receiving a discharge opioid prescription with greater than 50 OME per day before and after release of the CDC opioid guideline. Data are presented as the percentage of postoperative patients receiving a postoperative discharge opioid prescription with a daily dose greater than 50 OME in quarterly intervals from the 1^st^ quarter of 2013 through the 4^th^ quarter of 2019. The vertical line represents the first quarter after the release of the CDC guideline in March 2016. By the 2^nd^ quarter of 2018, the proportion of postoperative patients receiving a discharge opioid prescription with greater than 50 daily OME had decreased to less than 50%. Dc = discharge; Rx = prescription, and CI = confidence interval

The mean daily dose prescribed pre-guideline decreased by 0.387 OME per quarter (95% CI, -0.661 to -0.112) (Fig. 2a). Post-guideline, there was a significant change in the trend of mean daily dose prescribed by 2.307 OME per quarter (95% CI, -2.560 to -2.055, *p* < 0.0001). Overall, the mean daily dose prescribed for patients decreased by 46.5%, from 77.18 ± 3.17 OME in the 1^st^ quarter of 2016 to 41.28 ± 1.16 OME in the last quarter of 2019 (*p* < 0.0001).

The proportion of patients with a daily dose of discharge opioid prescription greater than 50 OME decreased by 0.413 percentage points per quarter (95% CI, -0.768% to -0.057%) (Fig. 2b). Post-guideline, the trend changed significantly, decreasing by 1.814 percentage points per quarter (95% CI, -2.141 to -1.488%, *p* < 0.0001). Overall, the percentage of patients with a discharge opioid prescription greater than 50 OME per day decreased by 45.0%, from 57.0% in the 1^st^ quarter of 2016 to 31.4% in the last quarter of 2019 (*p* < 0.0001).

### Trend change in total dose of postoperative discharge opioid prescriptions after CDC guideline

**Fig. 3:**
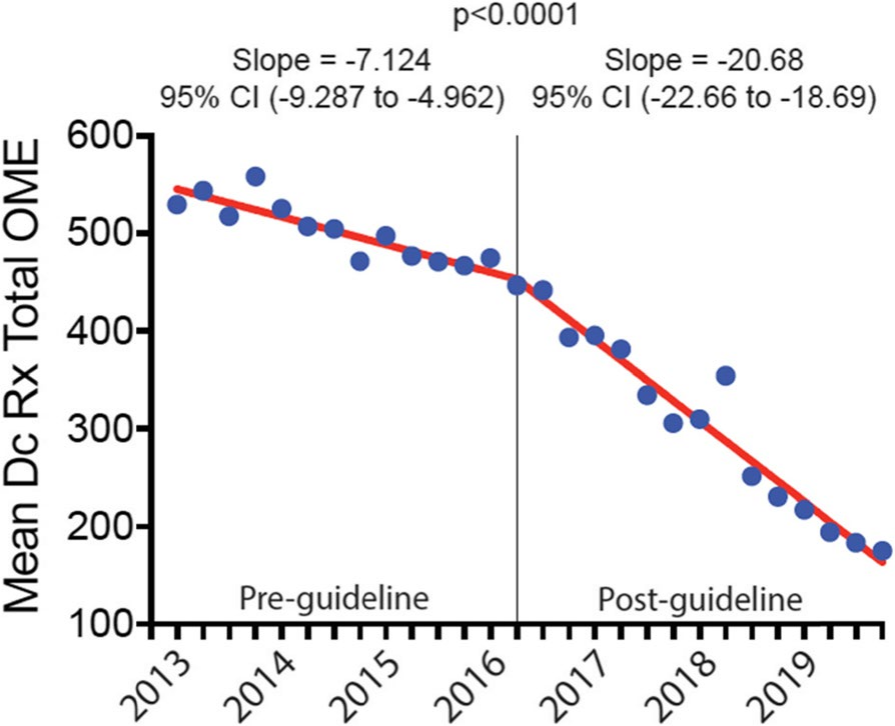
Total dose prescribed on the postoperative discharge opioid prescription, 2013–2019. Mean total dose prescribed on the postoperative discharge opioid prescription before and after the release of the CDC opioid guideline. Data are presented as the mean total dose prescribed on the postoperative discharge opioid prescriptions in quarterly intervals from the 1^st^ quarter of 2013 through the 4^th^ quarter of 2019. The vertical line represents the first quarter after the release of the CDC guideline in March 2016. Dc = discharge; Rx = prescription, and CI = confidence interval

The mean total dose prescribed pre-guideline decreased by 7.124 OME per quarter (95% CI, -9.287 to -4.962) (Fig. 3). There was a significant trend change in the post-guideline period, with a decrease of 20.68 OME per quarter (95% CI, -22.66 to -18.69, *p* < 0.0001). Overall, the total dose prescribed for patients decreased 63.7%, from 474.61 ± 18.96 OME in the 1^st^ quarter of 2016 to 175.27 ± 7.54 OME in the last quarter of 2019 (*p* < 0.0001).

### Trend change in 30-day postoperative opioid refills after CDC guideline

**Fig. 4:**
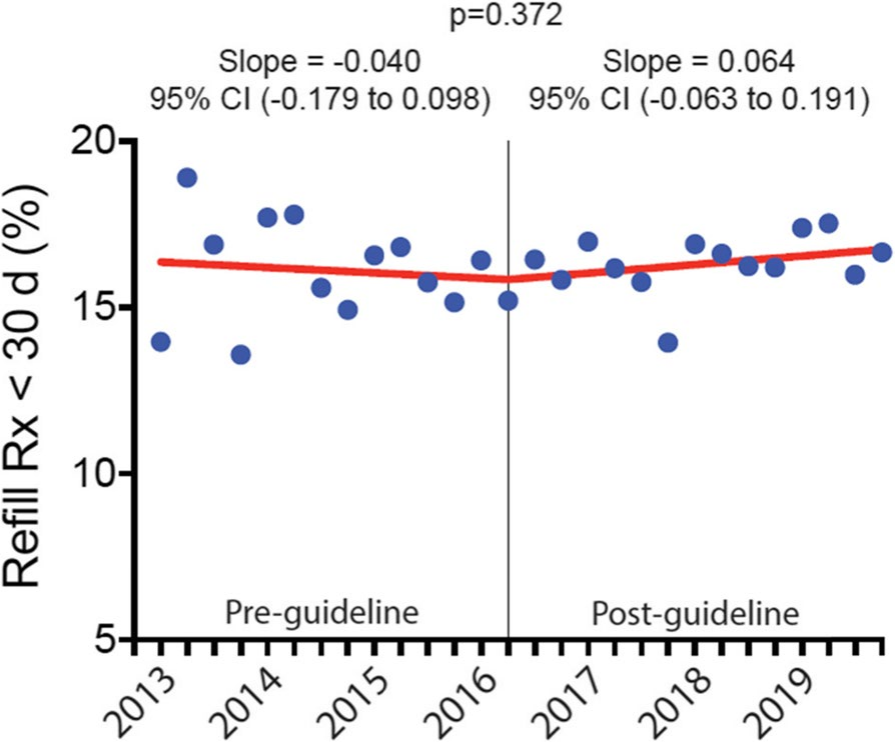
Proportion of postoperative patients receiving an opioid refill prescription within 30 days of discharge, 2013–2019. Proportion of postoperative patients receiving an opioid refill prescription within 30 days of discharge before and after the release of the 2016 CDC opioid guideline. Data are presented as the percentage of postoperative patients receiving an opioid refill prescription within 30 days of discharge in quarterly intervals from the 1^st^ quarter of 2013 through the 4^th^ quarter of 2019. The vertical line represents the first quarter after the release of the CDC guideline in March 2016. Rx = prescription, and CI = confidence interval

Opioid refill prescriptions within 30 days of discharge remained stable throughout the study, with no statistically significant change in quarterly refill trends (-0.040 percentage points per quarter, 95% CI, -0.179 to 0.098 pre-guideline versus 0.064 percentage points per quarter, 95% CI, -0.063 to 0.191 post-guideline, *p* = 0.372) or in the proportion of patients receiving a refill prescription (16.41% in the 1^st^ quarter of 2016 versus 16.65% in the last quarter of 2019, *p* = 0.865) (Fig. 4).

### Sensitivity analysis

Due to the change in volume of obstetrics and gynecology procedures at our institution during the study period, we performed the same ITSA analyses on our obstetrics and gynecology cases, the results of which did not differ from overall trends (not shown).

## Discussion

In this interrupted time series analysis, we found that the publication of the 2016 CDC opioid guideline was associated with significant trend changes in opioid prescribing practices for postsurgical patients. By assessing the association of provider prescribing practices with CDC guideline release, our study adds additional perspective to prior work that focused primarily on opioids filled by surgical patients after CDC guideline release [14, 16]. Specifically, we identified significant decreases in the day’s supply, daily dose, and total dose of opioid prescriptions written at discharge. Compared to the 1^st^ quarter of 2016 when the CDC guideline was released, the mean days’ supply, mean daily dose and mean total dose on the discharge opioid prescription decreased by 49.5%, 46.5%, and 63.7%, respectively, by the 4^th^ quarter of 2019.

The CDC guideline recommends prescribing up to three days’ supply for acute non-surgical pain indications and a starting daily dose of less than 50 OME to reduce the risk of fatal overdose or other opioid-related adverse effects, including the risk of transitioning to chronic opioid use [12]. Although the CDC guideline does not apply to surgical patients [12], we found that the mean days’ supply had decreased to less than 3 days by the 3^rd^ quarter of 2019, and the mean daily dose had decreased to less than 50 OME by the 1^st^ quarter of 2019 at our institution, a tertiary care hospital performing complex surgical procedures in patients with a range of comorbid conditions.

Importantly, despite the significant decreases in the OME and days’ supply of the discharge opioid prescriptions for surgical patients after the release of the 2016 CDC guideline, the percentage of patients requiring opioid refill prescriptions within 30 days of discharge did not change when compared with the pre-guideline period. This suggests that the significant reduction in the discharge opioid prescription did not result in insufficient opioid coverage for surgical patients. Our findings are consistent with previous reports that the probability of having an opioid refill within 30 days after surgery was not correlated with the dosage of the discharge opioid prescription [17], and that reducing the amount of opioid on the discharge prescription did not compromise post-operative pain control or patients’ satisfaction [16, 18–20].

Although our results suggest that the 2016 CDC’s opioid guideline of less than 3 days’ supply and less than 50 daily OME might be applied to opioid prescriptions for certain surgical patients, we caution against extrapolating the CDC guideline to all surgical settings. While the mean daily dose and the mean days’ supply did fall below the CDC recommendations by 2019, there was still a wide range of discharge daily doses and days’ supply represented in our study. For example, by the end of 2019, 39.8% of discharge opioid prescriptions still exceeded a 3-day supply, and 31.4% of prescriptions had a daily dose of greater than 50 OME. It has been previously reported that the dosage of the discharge opioid prescription closely correlates with surgical complexity, and the opioid requirement for patients after different types of surgical procedures can vary [21, 22]. In addition, setting a simple limit for opioid prescriptions to surgical patients without the ability to individualize the dosage has been shown to be problematic [23]. A better approach might be to evaluate pre-discharge inpatient opioid consumption to guide the opioid prescription at discharge [24, 25]. Additional studies will be needed to determine the appropriate indications for applying the current CDC opioid guideline to the discharge opioid prescription to surgical patients. Further studies will also be needed to follow the trend of opioid prescriptions for surgical patients after 2019 to determine the appropriate range of dose and duration without increasing opioid refill prescriptions.

Our study was conducted during a time of increasing evidence of harm associated with unsafe opioid prescribing practices, the introduction of DEA rescheduling of hydrocodone containing drugs, and the introduction of institutional opioid prescribing initiatives, which could have contributed to safer opioid prescribing practices. While California, where our study is located, did not pass state-specific legislation to limit opioid prescriptions during the study period, the state did update its prescription drug monitoring programs (PDMP) policy in July 2016 requiring mandatory registration of opioid prescribers, when previously this had been voluntary [26]. As an institution, surgery specific care protocols like enhanced recovery after surgery (ERAS) were adopted both before and after the 2016 CDC guideline was released, some of which included guidance on appropriate dosing of opioid prescriptions at discharge. Nevertheless, ERAS initiatives were introduced over multiple years, both before and after the March 2016 CDC guideline release rather than being implemented at a single point in time. These opioid-related initiatives could have directly or indirectly impacted our institution’s overall opioid prescribing trends. Regardless, we found a statistically significant accelerated rate of decline in several key opioid prescribing indicators after March 2016. Given the March 2016 inflection point, these findings suggest that the release of the CDC guideline may explain the changes in the opioid prescriptions written for surgical patients at our institution.

This study has some limitations. The study was conducted at a single academic medical center in opioid-naïve surgical patients requiring an inpatient post-operative stay. Therefore, our findings may not be representative of prescribing practices at non-academic hospitals, among patients undergoing outpatient surgeries, or among patients who used opioids prior to admission. In addition, since our study was an analysis assessing discharge opioid prescriptions across all inpatient procedures at our academic medical center, the results may not reflect the trends of individual surgical subspecialties or surgical procedures; analysis of these individual surgical subspecialty trends is currently underway. Furthermore, because we did not analyze post-discharge pain scores, despite the lack of change in the rate of postoperative opioid refills, we cannot definitively state that patients’ postoperative pain remained unchanged during the study period. However, while insurance databases provide data on prescriptions filled, they do not include opioid prescriptions written by the provider. Therefore, analyzing institutional EMR data provides a reliable way to obtain information about opioid prescribing practices on a provider-level and can be used to detect changes in physician practice patterns in response to new guidelines.

In conclusion, we found that the release of the 2016 CDC opioid guideline was associated with a significant reduction in discharge opioid prescriptions without a concomitant increase in opioid refills within 30 days of discharge for surgical patients. By 2019, the mean postsurgical opioid prescription decreased to less than 3 days’ supply and less than 50 OME per day in our medical center.

## Supplementary Information

**Additional file 1: FigureS1.** Interrupted time series analysis with modeltesting beta 2 intercept. **SupplementalMaterial 1. **Interrupted Time Series Analysis [27].

## Data Availability

Raw large scale electronic medical record data were generated at our institution. The raw electronic medical record data were generated at our institution at the point of care during the usual course of providing healthcare and the data was de-identified prior to making it available for research. The datasets generated and analyzed during the current study are not publicly available because some of the covariates that were made available to the research team are still classified as protected health information by our IRB. Datasets can be made available from the corresponding author on reasonable request.

## Abbreviations

OME: Oral morphine equivalents
CDC: Centers for Disease Control
EMR: Electronic medical record
ITSA: Interrupted time series analysis
