# Supplementary material for: Opioid prescribing practices at hospital discharge for surgical patients before and after the Centers for Disease Control and Prevention’s 2016 opioid prescribing guideline
Source: BMC Anesthesiol. 2022 May 11;22:141. doi: 10.1186/s12871-022-01678-6 (PMC9097447; doi:10.1186/s12871-022-01678-6)
Supplement: Supplementary file 1 — Additional file 1: FigureS1. Interrupted time series analysis with modeltesting beta 2 intercept. SupplementalMaterial 1. Interrupted Time Series Analysis [27]. [file 12871_2022_1678_MOESM1_ESM.docx]

Supplementary Materials and Figures

**Figure S1: Interrupted time series analysis with model testing beta 2 intercept**

*
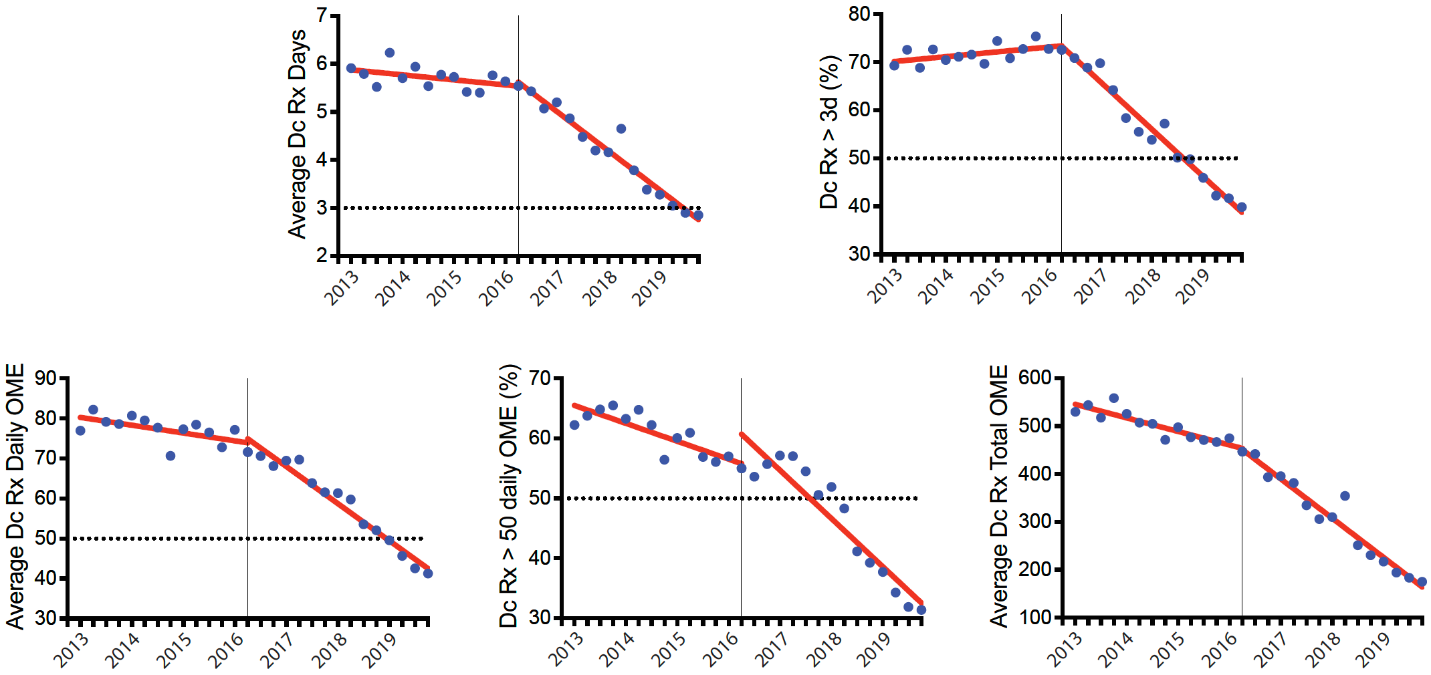
*

Data are presented in quarterly intervals on the x-axis. Vertical solid black lines represent the implementation of the 2016 CDC guidelines. DC = discharge, OME = oral milligram equivalents, Rx = prescription.

S1a: Changes in average length in days of the discharge prescription.

S1b: Proportion of patients receiving a discharge prescription greater than 3 days.

S1c: Changes in average daily OMEs on the discharge prescription.

S1d: Proportion of patients receiving a discharge prescription greater than 50 OMEs.

S1e: Changes in average total OMEs on the discharge prescription.

**Supplemental Material 1: Interrupted Time Series Analysis**

Data were analyzed by segmental linear regression with least squares regression (Prism 9.0) as an interrupted time series analysis. We followed the standard ITSA segmented regression model *Y_t_ = β_0_ + β_1_T_t_ + β_2_X_t_ + β_3_T_t_X_t_*,^27^ where *Y_t_* is the clinical outcome measured quarterly at timepoint t, T*_t_* is the time since the start of the study from the 1^st^ quarter of 2013, X*_t_* represents guideline release (i.e., the intervention) with pre-guideline quarters designated as 0 and post-guideline quarters designated as 1, and *T_t_X_t_* is the interaction term. *β_0_* is the intercept and represents the initial discharge characteristic at the 1^st^ quarter of 2013, *β_1_* is the slope in the pre-guideline period, and *β_3_* is the slope change in the post-guideline period. *β_2_* is the level change immediately post-guideline, defined as the immediate difference between the observed value at the end of the pre-guideline and beginning of the post-guideline periods. As the 2016 CDC opioid prescribing guideline made it clear that the recommendations in the guideline did not apply to opioid prescription to postsurgical patients, our null hypothesis was that the guidelines had no measurable effect on surgical prescribing. Accordingly, for the purpose of model building we set *β_2_* as 0, and our ITSA segmented regression model was simplified as *Y_t_ = β_0_ +β_1_T + β_3_TX_t_*. This hypothesis was empirically tested, and results are available in the appendix showing the model with the beta 2 intercept coefficient (Figure S1). We reported the slopes and their 95% confident intervals (CI) before and after the CDC opioid guideline was released, as well as the p- values to determine the model as segmental linear regression or as simple linear regression of a straight line.
